# Opposing roles of CXCR4 and CXCR7 in breast cancer metastasis

**DOI:** 10.1186/bcr3074

**Published:** 2011-12-09

**Authors:** Lorena Hernandez, Marco AO Magalhaes, Salvatore J Coniglio, John S Condeelis, Jeffrey E Segall

**Affiliations:** 1 Department of Anatomy and Structural Biology, Albert Einstein College of Medicine, 1300 Morris Park Avenue, Bronx, NY 10461, USA; 2 Gruss-Lipper Biophotonics Center, Albert Einstein College of Medicine, 1300 Morris Park Avenue, Bronx, NY 10461, USA

## Abstract

**Introduction:**

CXCL12-CXCR4 signaling has been shown to play a role in breast cancer progression by enhancing tumor growth, angiogenesis, triggering cancer cell invasion *in vitro*, and guiding cancer cells to their sites of metastasis. However, CXCR7 also binds to CXCL12 and has been recently found to enhance lung and breast primary tumor growth, as well as metastasis formation. Our goal was to dissect the contributions of CXCR4 and CXCR7 to the different steps of metastasis - *in vivo *invasion, intravasation and metastasis formation.

**Methods:**

We overexpressed CXCR4, CXCR7 or both in the rat mammary adenocarcinoma cell line MTLn3. Stable expressors were used to form tumors in severe combined immunodeficiency (SCID) mice, and *in vivo *invasiveness, intravital motility, intravasation, and metastasis were measured.

**Results:**

We found that CXCR4 overexpression increased the chemotactic and invasive behavior of MTLn3 cells to CXCL12, both *in vitro *and *in vivo*, as well as *in vivo *motility and intravasation. CXCR7 overexpression enhanced primary tumor growth and angiogenesis (as indicated by microvessel density and VEGFA expression), but decreased *in vivo *invasion, intravasation, and metastasis formation. *In vitro*, expression of CXCR7 alone had no effect in chemotaxis or invasion to CXCL12. However, in the context of increased CXCR4 expression, CXCR7 enhanced chemotaxis to CXCL12 but decreased invasion in response to CXCL12 *in vitro *and *in vivo *and impaired CXCL12 stimulated matrix degradation. The changes in matrix degradation correlated with expression of matrix metalloproteinase 12 (MMP12).

**Conclusions:**

We find that CXCR4 and CXCR7 play different roles in metastasis, with CXCR4 mediating breast cancer invasion and CXCR7 impairing invasion but enhancing primary tumor growth through angiogenesis.

## Introduction

There are currently two known receptors for CXCL12: CXCR4 and CXCR7 [1,2], which belong to the family of G-protein coupled receptors (GPCRs). CXCR4 is expressed in several human cancers including glioma [3], neuroblastoma [4], pancreatic [5] and breast [6], with overexpression of CXCR4 in breast cancer correlating with poor patient prognosis [7-9]. CXCL12/CXCR4 signaling has been reported to stimulate growth of several tumors including breast [10-13], with carcinoma-associated fibroblasts (CAFs) being an important source of CXCL12 in the tumor microenvironment [14]. CAFs can enhance tumor growth in a paracrine manner, with secreted CXCL12 directly stimulating growth of CXCR4 expressing breast cancer cells, and in an endocrine manner, recruiting endothelial progenitor cells (EPCs) to the primary tumors, thus enhancing angiogenesis [15]. CXCL12, also known as SDF-1, belongs to the CXC family of chemokines. CXCL12 functions as a growth factor for B cell progenitors [16], a chemotactic factor for both T cells and monocytes, a regulator of hematopoiesis and as a chemoattractant for tissue-committed stem cells [17,18]. Importantly, CXCL12 has been found to be expressed in many human solid tumors including breast, pancreas and prostate cancers, and glioblastoma [17], with high levels of CXCL12 expression correlating with poor prognosis of breast cancer patients [19].

CXCL12/CXCR4 signaling has been shown to stimulate the chemotactic and invasive behavior of breast cancer cells *in vitro *and *in vivo *[6,10,19-21], and has been proposed to serve as a homing mechanism for cancer cells to sites of metastasis. CXCL12 is expressed at high levels in the bone marrow, lung, liver, and lymph nodes, common sites of breast cancer metastasis, with protein extracts from these organs stimulating chemotaxis of breast cancer cells in a CXCR4-dependent manner [6]. Furthermore, downregulation of CXCR4 signaling using a neutralizing antibody or miRNA, decreases spontaneous and experimental lung metastasis formation of MDA-MB-231 cells [6,20].

Like CXCR4, CXCR7 is also expressed in different human cancers, including breast, being highly expressed in the tumor vasculature [22,23]. CXCR7 is considered an atypical GPCR because ligand binding does not result in intracellular Ca^2+ ^release [2,24], and there are conflicting reports on the ability of CXCR7 to activate phosphatidylinositol 3-kinase (PI3K) or mitogen-activated protein kinase (MAPK) signaling, and to promote cell motility. Binding of CXCL12 or interferon-inducible T-cell alpha chemoattractant (I-TAC/CXCL11), the other known CXCR7 ligand, to CXCR7 activates PI3K and MAPK signaling in astrocytes, Schwann cells, gliomas, rhabdomyosarcoma, and pancreatic cancer cells [23-26]. Moreover, CXCR7 has been reported to mediate CXCL12 chemotaxis in T cells [1] and rhabdomyosarcoma cells [26], and to promote hepatocellular carcinoma invasion *in vitro *[27]. However, other studies have shown that CXCR7 does not play a role in bare filter migration but in transendothelial migration [28], and that CXCR7 plays no role in T cell chemotaxis or MAPK/PI3K signaling [29]. Although the interaction of CXCR7 with G proteins is controversial, new studies have found that CXCR7 binds to β-arrestin 2, with this interaction resulting in receptor internalization [28,30,31], and mediating chemotaxis to I-TAC in vascular smooth muscle cells [32]. Furthermore, CXCR4 and CXCR7 can form both homodimers and heterodimers with heterodimer formation suggested to modulate CXCR4 signaling both positively, and negatively [33-35]. Most recently, CXCR4+CXCR7+ MDA MB 231 cells have been shown to chemotax in response to CXCL12 stimulation better than 231 cells expressing only CXCR4, with this chemotactic response being dependent on β-arrestin 2 [36].

CXCR7 has been implicated in enhancing cancer cell adhesion to fibronectin and endothelial cells [2,23,27]; increasing cell survival by decreasing apoptosis [2,23] and promoting primary tumor growth of lymphoma, lung, breast, prostate and hepatocellular cancer cells [2,22,23,27]. CXCR7 expression has been reported to contribute to tumor angiogenesis through the secretion of angiogenic factors such as vascular endothelial growth factor (VEGF) [23,27], as well as to promote experimental metastasis formation of breast cancer cells [22].

Although CXCL12 signaling has been implicated in breast cancer metastasis as a homing mechanism for cancer cells to common sites of metastasis, not much is currently known about the role of CXCL12 signaling in the early steps of metastasis within the primary tumor. Also, the role of CXCR7 in breast cancer cell motility, tumor growth and metastasis is still unclear, with the effect of coexpression of CXCR4 and CXCR7 in these processes mostly unknown. With research suggesting that both CXCR4 and CXCR7 alone can enhance metastasis, we set out to dissect the roles of CXCR4 and CXCR7 in the different steps of metastasis (invasion, intravasation, and metastasis formation) by overexpressing CXCR4, CXCR7, or both receptors in the rat mammary adenocarcinoma cell line MTLn3. Here we report that CXCR4 overexpression increases the chemotactic and invasive behavior of MTLn3 cells, *in vitro *and *in vivo*, to CXCL12, as well as their motile behavior within the primary tumor. Furthermore, although CXCR4 overexpression had no effect on primary tumor growth, it enhanced intravasation without affecting spontaneous lung metastasis formation. CXCR7 overexpression alone did not result in CXCL12-induced chemotaxis or invasion *in vitro*; however, in the context of high CXCR4 expression it further increased the *in vitro *chemotactic response of MTLn3 CXCR4 cells to CXCL12, while reducing invasion and matrix degradation. *In vivo*, CXCR7 increased primary tumor growth while it impaired invasion to CXCL12, intravasation and spontaneous lung metastasis formation. CXCR7 overexpression downregulated the effects of CXCR4 in motility within the primary tumor, intravasation, and spontaneous lung metastasis formation.

## Materials and methods

### Cell lines

All MTLn3 cell lines were grown in alpha MEM supplemented with 5% FBS (100-106; Gemini Bio-Products, West Sacramento, CA, USA) and 0.5% penicillin/streptomycin (15140-122; Invitrogen, Grand Island, NY, USA). To create the human CXCR4 expressors, hCXCR4 was transferred from the pDNR-Dual hCXCR4 vector (Harvard Institute of Proteomics, Boston, MA, USA), to the JP1520 retroviral vector following the Creator Cloning protocol, using Cre recombinase (Clontech, Mountain View, CA, USA) and Max Efficiency DH5alpha bacteria (Life Technologies, Grand Island, NY, USA) grown in 7% sucrose, 30 μg/ml chloramphenicol plates. Colonies were picked and correct insertion of human CXCR4 verified by sequencing. The human CXCR7 sequence was digested out from the pcDNA 3.1+ plasmid (kindly provided by ChemoCentryx, Mountain View, CA, USA) using NotI, the ends blunted using DNA Polymerase I, large Klenow fragment (NEB, Ipswich, MA, USA), to insert into JP1520, which was digested with BamHI and BbsI removing the loxP site, ends blunted as above and treated with Antarctic phosphatase (NEB, Ipswich, MA, USA). Both insert and vector were gel purified using the Qiagen Gel extraction kit, then ligated using a Rapid DNA Ligation kit (Roche, Branchburg, NJ, USA). Subcloning efficiency DH5alpha bacteria (Life Technologies, Grand Island, NY, USA) were transformed with the ligated vector and colonies screened for correct insertion of hCXCR7 using differential enzyme digestions, followed by verification using sequencing analysis. MTLn3-GFP (MTLn3 cells expressing green fluorescent protein) cells were transduced with either the empty JP1520 vector, JP1520-CXCR4, JP1520-CXCR7, or both CXCR4 and CXCR7, by first transfecting Phoenix packaging cells with 2 μg of each vector using lipofectamine (Invitrogen, Grand Island, NY, USA), collecting virus and transducing MTLn3-GFP cells seeded at 60% confluency. Transduced cells were selected with 1 μg/ml puromycin. MTLn3 CXCR7 and MTLn3 CXCR4-CXCR7 cells were subsequently fluorescence-activated cell sorting (FACS) sorted in a DakoCytomation MoFlo to obtain a homogenous population of CXCR7 expressing cells. MDA MB 435 cell lines were grown in DMEM (10-013 CV, Cellgro, Manassas, VA, USA) supplemented with 10% FBS (S11550, Atlanta Biologicals, Lawrenceville, GA, USA) and 0.5% penicillin/streptomycin. 435 cells seeded at 60% confluency, were transduced with either the empty JP1520 vector, JP1520-CXCR4 or JP1520-CXCR7 using premade virus, with transductants selected using 1 μg/ml puromycin. MDA MB 435 CXCR7 cells were FACS sorted in a DakoCytomation (Carpinteria, CA, USA) MoFlo to obtain a homogenous population of CXCR7 expressing cells. The 435 double overexpressors, CXCR4-CXCR7, were made by transducing sorted 435-CXCR7 cells with JP1520-CXCR4 virus and then FACS sorted for high CXCR4 expression. MDA MB 435 CXCR4 cells were sorted at the same time to obtain cell lines with homogenous CXCR4 expression.

### Reverse transcription and PCR

MTLn3 cells grown to 70 to 85% confluency were used for RNA isolation using the Qiagen RNeasy Mini kit with DNase I treatment (Valencia, CA, USA). A 1 μg sample of total RNA was used for reverse transcription using Superscript III and random hexamers in a 20 μl reaction volume. A 2 μl aliquot of the reaction was used for PCR using Taq polymerase for 30 cycles. Primers used were: rat glyceraldehyde 3-phosphate dehydrogenase (GAPDH) (PPR06557A Superarray), rat CXCR4 (5' AGGAACTGAACGCTCCAGAA 3' and 5' AACCACACAGCACAACCAAA 3'), human CXCR4 (5' CTCCAAGCTGTCACACTCCA 3' and 5' TCGATGCTGATCCCAATGTA 3'), human and rat CXCR7 (5' GCACTACATCCCGTTCACCT 3' and 5'AAGGCCTTCATCAGCTCGTA 3'). PCR products were run in a 1.5% agarose gel containing ethidinium bromide. For quantitation of endogenous expression of rat CXCR4, the collected cDNA was used for quantitative real-time PCR with SYBR Green (PA-012, SuperArray Biosciences, Frederick, MD, USA) and an Applied Biosystems 7900HT (Carlsbad, CA, USA). To evaluate the expression of matrix metalloproteinases (MMPs) in the different MTLn3 transductants, the cell lines were grown to 80 to 90% confluency in six-well plates, starved overnight in alpha-MEM/0.35% BSA in a 37°C incubator, and then stimulated for four hours with 10 nM CXCL12 (460-SD; R&D systems, Minneapolis, MN, USA) in alpha-MEM/0.35% BSA or just alpha-MEM/0.35% BSA at 37°C. RNA was extracted using the Qiagen RNeasy Mini kit with DNase I treatment. A 2 μg sample of total RNA was used for reverse transcription using a Superscript First Strand kit (11904-018, Invitrogen, Grand Island, NY, USA) and cDNA used for real time PCR with SYBR Green (PA-012) on an Applied Biosystems 7900HT. Rat specific primers for MMPs were obtained from real-time primers (Elkins Park, PA, USA). RNA expression of MMPs was normalized to GAPDH.

### Fluorescent activated cell sorting (FACS)

To analyze the levels of CXCR4 and CXCR7 expression in the MTLn3 and MDA MB 435 cell lines, cells were grown to 80% confluency, detached at 37°C using PBS without Ca^2+^/Mg^2+ ^+2 mM ethylenediaminetetraacetic acid (EDTA) and resuspended in 1 ml cold PBS without Ca^2+^/Mg^2+ ^supplemented with 0.2% BSA. Cells were labeled with either control mouse IgG antibody (MAB002; R&D systems, Minneapolis, MN, USA), anti-human CXCR4 antibody (MAB172; R&D systems, Minneapolis, MN, USA) or anti-human CXCR7 antibody (11G8; ChemoCentryx, Mountain View, CA, USA) for 45 minutes at 4°C. Unbound primary antibody was removed by washing and bound antibody was detected with APC antimouse secondary antibody (Jackson ImmunoResearch, West Grove, PA, USA). Expression of rat CXCR4 protein was evaluated in the MTLn3 transductants using a rat specific CXCR4 antibody (ab7199, Abcam, Cambridge, MA, USA) with a rabbit IgG as a control (011-000-003, Jackson ImmunoResearch, West Grove, PA, USA) and anti-rabbit DyLight 649 as the secondary antibody (111-496-144, Jackson ImmunoResearch, West Grove, PA, USA). Fluorescently labeled cells were evaluated using a Becton Dickinson LSRII (Franklin Lakes, NJ, USA). FCS files were analyzed using FlowJo software (Ashland, OR, USA).

### In vitro chemotaxis

Chemotaxis was evaluated using a 48-well microchemotaxis chamber (Neuroprobe, Gaithersburg, MD, USA) and PVP-free 8 μm pore polycarbonate filters (Neuroprobe, Gaithersburg, MD, USA) coated with 27 μg/ml rat tail collagen type I (BD Bioscience, Franklin Lakes, NJ, USA). Cell lines were starved in L15 medium supplemented with 0.35% BSA for three hours at 37°C, detached using PBS without Ca^2+^/Mg^2+ ^+ 2 mM EDTA and resuspended in L15-0.35% BSA to plate 2 × 10^4 ^cells per well for MTLn3 transductants, or 1.5 × 10^4 ^cells per well for the MDA MB 435 transductants. CXCL12 solutions were prepared in L15-0.35% BSA and placed in the bottom wells with cells plated in the top wells of the assembled chamber. To inhibit CXCL12 binding to CXCR7, we added 10 nM I-TAC (572-MC; R&D systems, Minneapolis, MN, USA), or used the CXCR7 inhibitors CCX733 (ChemoCentryx, Mountain View, CA, USA) and CCX771 (ChemoCentryx, Mountain View, CA, USA) added to both top and bottom wells, including vehicle (DMSO) as a control. To inhibit CXCR4, we added AMD3100 (Sigma, St. Louis, MO, USA) to both top and bottom wells. After a four-hour incubation at 37°C, filters were placed in 10% formalin solution to fix the cells for 30 minutes, cells on the top of the filter, non-migrating cells, were removed using a cotton swab, and migrating cells subsequently stained overnight in hematoxylin. The number of cells crossing the filter in one representative 10× field was counted per well for the MTLn3 transductants, using a Nikon Labophot light microscope (Melville, NY, USA), and corresponding wells averaged per experiment. To determine MDA MB 435 chemotaxis, the number of cells that crossed each well were counted and corresponding wells averaged per experiment.

### In vitro invasion

MTLn3 transductants grown to 70 to 85% confluency were starved for three hours in alpha-MEM supplemented with 0.35% BSA in a 37°C incubator. Cells were detached using PBS without Ca^2+/^Mg^2+ ^containing 2 mM EDTA, resuspended in alpha-MEM supplemented with 0.35% BSA to plate 1 × 10^5 ^cells in a 500 μl volume on top of Matrigel-precoated 8 μm pore-filters (354480; BD Biosciences transwells, Franklin Lakes, NJ, USA) that had been equilibrated for one hour with alpha-MEM/0.35% BSA in a 37°C incubator. Cells were allowed to invade overnight in a 37°C incubator in response to either alpha-MEM/0.35% BSA alone or containing 10 nM CXCL12. The filters were fixed in 10% formalin for 30 minutes and stained with crystal violet for 15 minutes. Cells that had not invaded were removed with a cotton tip applicator from the top of the filter, filters removed, placed in a coverslip and the total number of invading cells present in a filter counted using a Nikon Labophot light microscope with a 10× objective.

### Matrix degradation assay

MTLn3 CXCR4 or MTLn3 CXCR4-CXCR7 GFP labeled cells were plated overnight on MatTek dishes, at 1 × 10^5 ^cells/dish, over a thin Alexa 405-gelatin matrix in the presence of the protease inhibitor GM6001 (10 μm). Cells were subsequently starved for three hours, washed three times in starvation media [37] and stimulated with 5 nM CXCL12 for six hours. At the end of the incubation time, cells were fixed in 3.7% paraformaldehyde and imaged. The images were processed using the ImageJ Spot enhancing filter 2D (3.0 pixels Gaussian filter) and the threshold levels set to select only degradation areas. The degradation area was normalized to the cell coverage area in the GFP channel. Alexa405 (A30000, Invitrogen, Grand Island, NY, USA) was conjugated to gelatin (G2500, Sigma, St. Louis, MO, USA) and thin matrix Alexa405-gelatin matrix was prepared as previously described [38]. Results are reported as the degraded area/cell area per field normalized to the MTLn3 CXCR4 unstimulated levels.

### In vivo invasion

All animal procedures were conducted observing the National Institutes of Health regulations on the use and care of experimental animals. Our animal protocol was approved by the Albert Einstein College of Medicine animal use committee. Female severe combined immunodeficiency (SCID) mice aged four to seven weeks from NCI were used for all experiments. To form primary tumors, MTLn3 transductants grown to 70 to 85% confluency were detached using PBS without Ca^2+^/Mg^2+ ^+ 2 mM EDTA, resuspended in cold PBS supplemented with 0.2% BSA to inject 5 × 10^5 ^cells per animal in a 100 μl volume. Cells were injected into the fourth mammary fat pad and tumors allowed to grow until they reached an average volume of 1,300 mm^3 ^for the *in vivo *invasion assay. Mice were anesthetized using isoflurane and blocking needles placed into the primary tumors using micromanipulators (MN-151; Narishige, East Meadow, NY, USA). Hamilton 33 gauge needles were loaded with a mixture of EDTA, 10% matrigel and CXCL12 dissolved in L15-0.35% BSA, and these experimental needles used in place of the blocking needles after the animal was appropriately setup. Detailed information about this assay can be found in [39]. To block the colony stimulating factor 1 (CSF-1) receptor on the mouse tumor associated macrophages we used the anti-mouse CSF-1R antibody (AFS98) [40] at 15 μg/ml; to block epidermal growth factor (EGF)-derived from the mouse tumor associated macrophages from binding to EGF receptor (EGFR) we used a neutralizing EGF antibody at a concentration of 20 μg/ml (AF2028; R&D systems, Minneapolis, MN, USA). An isotype IgG antibody (012-000-007 or 111-005-144, Jackson ImmunoResearch, West Grove, PA, USA) was used at the same concentration as the blocking/neutralizing antibodies as a control. To inhibit CXCR4 we used 100 nM AMD3100. Cells were allowed to invade into the needles for four hours and the contents of the needles were subsequently extruded into coverslips, invasive cells stained with 4',6-diamidino-2-phenylindole (DAPI) and counted using an Inverted Olympus IX70 microscope (Center Valley, PA, USA).

### Intravital imaging

Primary tumors of an average volume of 1,300 mm^3 ^were used for intravital imaging. Mice were anesthetized with isoflurane and a skin-flap surgery carefully performed to expose the primary tumor while minimizing damage to tissue and blood vessels. Animals were placed on the stage of an inverted microscope and the GFP-labeled carcinoma cells imaged using an Olympus Fluoview FV1000-MPE microscope (Center Valley, PA, USA) at an excitation of 880 nm with a 25 × 1.05 NA water objective. Collagen fibers were visualized by second harmonic generation. Time-lapse Z-series were taken at 5 μm steps for a total of 100 μm into the tumor over 30 minutes at two-minute intervals. Movies were analyzed using Image J http://imagej.nih.gov/ij/. A cancer cell was considered to be motile when it had protruded/translocated at least half a cell length, and the total number of motile cancer cells in a 50 μm Z-stack time-lapse movie was determined. A more detailed description of this protocol can be found in [41].

### Spontaneous metastasis and intravasation

Primary tumors were allowed to grow until they reached an average volume of 1,500 mm^3 ^to perform end-point metastasis assays. At this time, mice were anesthetized using isoflurane and blood collected via cardiac puncture from the right side of the heart to obtain cancer cells that had intravasated. The blood drawn was then plated into a 10 cm dish with alpha MEM supplemented with 5% FBS/0.5% P/S, and cancer cell colonies allowed to grow for a week in a 37°C incubator followed by counting using a light microscope. Tumor blood burden is reported as the total number of cancer cell colonies present in a dish normalized to the volume of blood plated. Lungs and primary tumors were harvested and fixed in 10% formalin solution. The lungs were paraffin-embedded, sectioned and stained with H&E to count the number of lung metastasis present in all lobes of a single section using a light microscope with a 10× objective. To evaluate lymph node metastasis, both axillary and inguinal lymph nodes were removed from tumor-bearing mice, fixed in 10% formalin solution, paraffin-embedded, sectioned and stained with H&E. The presence of metastases was assessed using a Nikon Labophot light microscope (Melville, NY, USA). To estimate bone marrow metastasis, the femur ipsilateral to the site of primary tumor growth was dissected and bone marrow was flushed using 1 ml syringes with 25-gauge needles into a 10 cm plate containing alpha MEM supplemented with 5% FBS/0.5% P/S. Plates were incubated at 37°C for a week and tumor colonies then counted.

### Immunohistochemistry

For microvessel density evaluation, formalin-fixed, paraffin-embedded sections from MTLn3 JP, MTLn3 CXCR4, MTLn3 CXCR7, and MTLn3 CXCR4-CXCR7 primary tumors were deparaffinized, rehydrated, blocked in donkey serum and stained with rat antimouse CD34 antibody (CL8927AP; Cedarlane labs, Burlington, NC, USA) at a 1:400 dilution for one hour. Slides were washed and subsequently stained with a biotinylated antirat secondary antibody for 50 minutes. The slides were rinsed and exposed to ABC-HRP (PK-6100, Vector, Burlingame, CA, USA) for 20 minutes, washed and exposed to diaminobenzidine (DAB) for one to four minutes (SK-4100, Vector, Burlingame, CA, USA), and subsequently counterstained with Harris hematoxylin (s212, Poly-scientific, Bay Shore, NY, USA), rinsed and mounted. Mean vessel density was determined by counting the number of blood vessels present per field seen in a light microscope using a 10× objective. A total of three different primary tumors were used per cell line, counting five fields per tumor. For VEGFA evaluation, samples for immunohistochemistry (IHC) were sectioned at 5 μm, deparaffinized in xylene followed by graded alcohols. Antigen retrieval was performed in 10 mM sodium citrate buffer at pH 6.0, heated to 96C, for 20 minutes. Endogenous peroxidase activity was quenched using 3% hydrogen peroxide in PBS for 10 minutes. Blocking was performed by incubating sections in 5% normal donkey serum with 2% BSA for one hour. The primary antibody to VEGFA, (PAB12284, ABNOVA, Walnut, CA, USA) was used at 1:250 for 1.5 hours at room temperature. The primary species (rabbit IgG) was substituted for the primary antibody to serve as a negative control. The sections were stained by routine IHC methods, using HRP rabbit polymer conjugate (Invitrogen, Grand Island, NY, USA), for 20 minutes to localize the antibody bound to antigen, with diaminobenzidine as the final chromogen. All immunostained sections were lightly counterstained with hematoxylin.

### Statistical analysis

Analysis of variance (ANOVA) was used to demonstrate that there were significant differences between conditions when there were more than two conditions, and paired analyses were performed using either student t-test, or Mann-Whitney test in order to identify the conditions that were significantly different. Correlation of MMP12 expression with CXCR4 and CXCR7 was performed using Oncomine with the following databases: Bittner Breast, Bonnefoi Breast, Desmedt Breast, Ginestier Breast, Gluck Breast, Hess Breast, Ivshina Breast, Loi Breast, and van't Veer Breast. SPSS was used to determine correlation coefficients and their significance from the downloaded expression data. The Oncomine database was also used to identify clinical parameters with which MMP12 mRNA levels were significantly correlated. The following parameters were examined: estrogen receptor (ER) positive, triple negative, high grade, metastasis, recurrence and survival. The probabilities of overexpression or underexpression of MMP12 for all breast cancer datasets containing more than 40 samples for which the parameters were provided by Oncomine were downloaded. For each parameter, the number of datasets in which the probability of MMP12 overexpression or underexpression was less than 0.05 was identified and the binomial cumulative probability distribution was used to determine the likelihood of that number occurring by chance using SPSS (IBM, Armonk, NY).

## Results

### CXCR7 enhances *in vitro *chemotaxis to CXCL12 in the presence of high CXCR4

**Figure 1:**
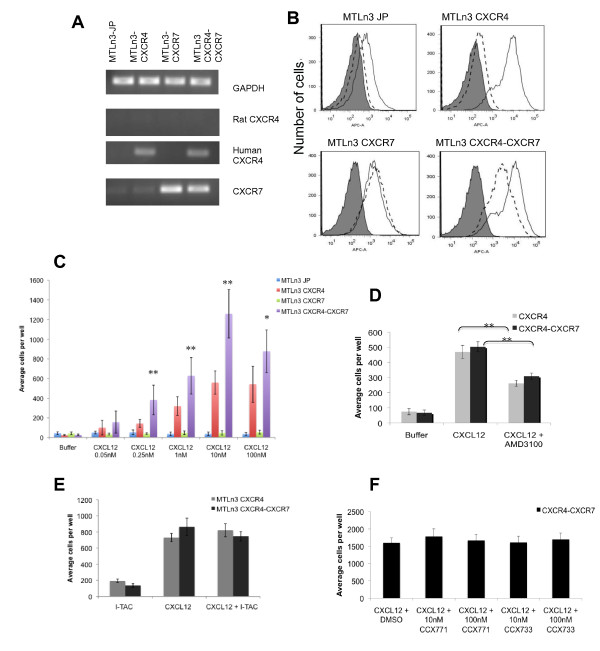
**Expression of CXCR7 increases the *in vitro *chemotactic response of MTLn3 CXCR4 cells to CXCL12**. **(a) **RT-PCR of RNA isolated from the indicated transductants using primers specific for either rat GAPDH, rat CXCR4, human CXCR4, or both rat and human CXCR7. **(b) **FACS analysis of the transductants. Representative FACS plots show receptor expression at the cell membrane: isotype control mouse IgG (grey shaded peaks), anti-CXCR4 antibody (solid lines, MAB172) and anti-CXCR7 antibody (dashed lines, 11G8). **(c) **Chemotaxis of transduced cell lines to CXCL12. Cells were allowed to chemotax for four hours at 37°C in a microchemotaxis chamber. Total number of cells per well are reported (11 to 33 wells were counted per condition). Comparison of MTLn3 CXCR4 chemotaxis to CXCL12 with that of MTLn3 JP cells shows a statistically significant increase at 0.25 nM, 1 nM, 10 nM, and 100 nM with a *P *value less than 0.005 as determined by t-test, and *P *= 0.079 at 0.05 nM CXCL12. Comparison of the chemotaxis of the double expressors, CXCR4-CXCR7, with that of MTLn3 JP cells show similarly statistically significant differences as determined by t-test, with *P *< 0.05 at 0.05 nM CXCL12 and *P *< 0.005 at the other concentrations. Statistically significant differences between MTLn3-CXCR4 and MTLn3 CXCR4-CXCR7 are indicated in the figure with *P *< 0.05 indicated by * and *P *< 0.005 indicated by **. **(d) **Chemotaxis of MTLn3 CXCR4 and MTLn3 CXCR4-CXCR7 cells to 1 nM CXCL12 with or without 100 nM AMD3100 (6 to 22 wells were counted per condition), *P *< 0.005 is represented by **. **(e) **Chemotaxis of MTLn3 CXCR4 and MTLn3 CXCR4-CXCR7 cells to 1 nM CXCL12 with or without 10 nM I-TAC (five to seven wells were counted per condition). **(f) **MTLn3 CXCR4-CXCR7 chemotaxis to 1 nM CXCL12 in the presence of vehicle DMSO, CCX771, or CCX733 (11 to 15 wells were counted per condition). Means and SEMs are shown.

To evaluate the roles of CXCR4 and CXCR7 in CXCL12-induced chemotaxis *in vitro*, the human open reading frames of these receptors were stably overexpressed in the rat mammary adenocarcinoma cell line MTLn3 using retroviral expression vectors. RT-PCR of these cell lines demonstrates clear increases in mRNA for the corresponding receptors (Figure 1a). A low level of endogenous rat CXCR4 mRNA expression in the MTLn3 cell line was confirmed by quantitative real-time PCR showing a C_t _of 35 cycles (GAPDH C_t _of 16). The levels of expression of CXCR4 and CXCR7 at the cell membrane were subsequently evaluated using FACS analysis (Figure 1b). Staining with a control isotype antibody is represented by a grey shaded peak in all plots. The MTLn3 JP empty vector control cell line shows low levels of expression of CXCR4 (solid line) with little expression of CXCR7 (dashed line), consistent with the PCR data. The CXCR4 transductant, MTLn3 CXCR4, shows higher expression of CXCR4 (solid line), while the CXCR7 transductant, MTLn3 CXCR7, shows higher expression of the CXCR7 receptor (dashed line). The levels of both CXCR4 and CXCR7 expression in the double transductant, MTLn3 CXCR4-CXCR7, show increases comparable with the respective single transductants. Mean fluorescence intensity values for the different transductants are included in Additional data file 1. The observed low levels of endogenous rat CXCR4 expression in the MTLn3 transductants were confirmed using a different antibody against full length rat CXCR4 [see Additional data file 2]. Although there is a slight increase in the surface expression of CXCR4 in the CXCR7 cell lines compared with control JP lines (Figure 1b) [see Additional data file 1] this was not evident in the subsequent FACS analysis using the rat specific CXCR4 antibody [see Additional data file 2], and did not produce any increase in CXCL12-induced chemotaxis compared with the JP control line (see below). In summary, we were able to increase the expression of both CXCR4 and CXCR7 at least three fold over the basal expression levels.

Having engineered MTLn3 cell lines overexpressing either CXCR4, CXCR7, or both receptors, we compared their chemotaxis to CXCL12 using a microchemotaxis chamber (Figure 1c). The MTLn3 CXCR4 cell line showed significantly increased CXCL12-induced chemotaxis (*P *< 0.005) compared with the control cell line, MTLn3 JP. The MTLn3 CXCR7 cell line showed no chemotactic response to CXCL12 (*P *> 0.1). Furthermore, overexpression of both CXCR4 and CXCR7 resulted in a significantly increased chemotactic response to CXCL12 compared with that of the MTLn3 CXCR4 cell line (*P *< 0.005). This migration phenotype suggests that although CXCR7 alone does not mediate CXCL12-induced motility, in the presence of CXCR4, CXCR7 augments CXCL12-stimulated motility. This was confirmed in the cancer cell line MDA-MB-435 previously shown to express low endogenous levels of CXCR4 [42] [see Additional data file 2], which also showed the CXCR4-CXCR7 double overexpressors to have the most chemotaxis to CXCL12.

To test whether the increased motility observed in the MTLn3 CXCR4-CXCR7 cell line was dependent on CXCR4, we added the CXCR4 inhibitor, AMD3100, at a concentration that specifically inhibits CXCR4 without acting as an agonist of CXCR7 [43]. Addition of AMD3100 significantly decreased CXCL12-induced chemotaxis of both MTLn3 CXCR4 and MTLn3 CXCR4-CXCR7 cells (*P *< 0.005) (Figure 1d) suggesting that CXCR4 is important for CXCL12-induced chemotaxis in the double overexpressors. Addition of I-TAC (CXCL11), a chemokine that binds to CXCR7 [2,22], failed to act as a chemoattractant or impair CXCL12-induced chemotaxis for either MTLn3 CXCR4, or MTLn3 CXCR4-CXCR7 cells (*P *> 0.5) (Figure 1e). Similarly, addition of the CXCR7 inhibitors CCX771 and CCX733 did not inhibit chemotaxis to CXCL12 in the MTLn3 CXCR4-CXCR7 cells (*P *> 0.4) (Figure 1f). These results show that *in vitro*, CXCR7 alone does not mediate CXCL12-induced chemotaxis. However, when expressed in cells with high levels of CXCR4, CXCR7 augments CXCR4-mediated chemotaxis to CXCL12, in agreement with recent studies using MDA-MB 231 cells [36]. Importantly, the chemotaxis response of the double overexpressors, CXCR4-CXCR7, was impaired in the presence of CXCR4 inhibitor, AMD3100, but not upon addition of ITAC or the CXCR7 inhibitors CCX771 or CCX733 suggesting that binding of CXCL12 to CXCR4 but not CXCR7 is needed for chemotaxis to occur.

### CXCR7 inhibits invasion to CXCL12

**Figure 2:**
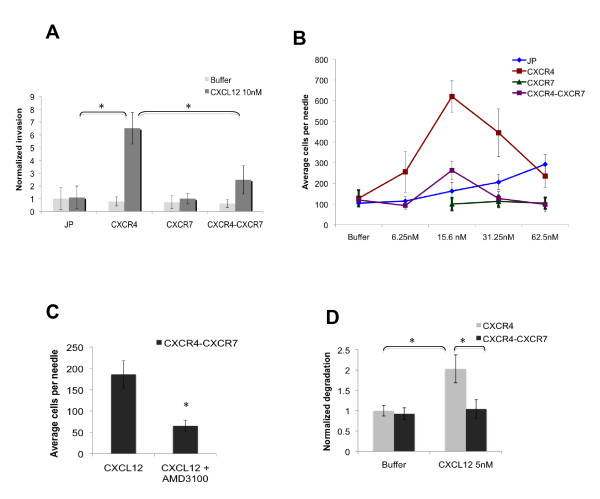
**CXCR4 expression enhances CXCL12 induced invasion, while CXCR7 expression impairs it**. **(a) ***In vitro *invasion of MTLn3 JP, MTLn3 CXCR4, MTLn3 CXCR7, and MTLn3 CXCR4-CXCR7 cells in the presence or absence of 10 nM CXCL12. The total number of invasive cells present per filter were counted and normalized to the invasive response of control MTLn3 JP cells in the absence of CXCL12 stimulation (*n *= 3 individual experiments per condition). **(b) ***In vivo *invasion of MTLn3 JP, MTLn3 CXCR4, MTLn3 CXCR7, and MTLn3 CXCR4-CXCR7 primary tumors in response to the indicated concentrations of CXCL12. Cells were allowed to invade for four hours into needles containing Matrigel plus or minus chemoattractant. Note that the buffer measurements for all transductants are plotted and overlap. For the MTLn3 CXCR7 transductant, there is no measurement at 6.25 nM CXCL12 as there were no responses at higher concentrations of CXCL12 and the chemotaxis data showed no response at low concentration of CXCL12. Data are from 10 mice with three to eight needles per condition for MTLn3 JP, 14 mice with 5 to 10 needles per condition for MTLn3 CXCR4, eight mice with three to six needles per condition for MTLn3 CXCR7, 13 mice with three to eight needles per condition for MTLn3 CXCR4-CXCR7. Comparison of MTLn3 CXCR7 invasion to CXCL12 with that of MTLn3 JP cells show statistically significant reduction at 31.25 nM (*P *< 0.05) and 62.5 nM (*P *< 0.005) using t-test. Comparison between MTLn3 CXCR4 and MTLn3 JP invasion shows statistically significant differences at 6.25 nM, 15.6 nM and 31.25 nM CXCL12 with *P *< 0.05, *P *< 0.005, and *P *< 0.05, respectively. MTLn3 CXCR4-CXCR7 tumors showed statistically significant decreased invasion at all concentrations of CXCL12 tested compared to MTLn3 CXCR4 6.25 nM *P *< 0.05, 15.6 nM *P *< 0.005, 31.25 nM *P *< 0.005, 62.5 nM *P *< 0.05. **(c) ***In vivo *invasion of MTLn3 CXCR4-CXCR7 tumors in response to 15.6 nM CXCL12 with or without 100 nM AMD3100. Three animals were tested with three to seven needles counted per condition. **(d) **Matrix degradation of MTLn3 CXCR4 and MTLn3 CXCR4-CXCR7 cells in the absence or presence of 5 nM CXCL12 (at least 19 fields were counted per condition). Means and SEMs are shown. *P *< 0.05 is represented by * as determined by t-test.

We next tested the role of these receptors in CXCL12-induced invasion *in vitro *using transwells precoated with Matrigel. Although overexpression of CXCR4, CXCR7, or both CXCR4 and CXCR7 did not affect MTLn3 basal invasion *in vitro *as measured in the presence of just buffer (*P *> 0.6) (Figure 2a, gray bars), CXCR4 expression enabled an invasive response of MTLn3 cells to CXCL12 *in vitro *(*P *< 0.05). CXCR7 expression alone failed to significantly enhance invasion to CXCL12 (*P *= 0.9). Surprisingly, in the context of CXCR4 overexpression, CXCR7 inhibited CXCL12-induced invasion compared with MTLn3 CXCR4 cells (*P *< 0.05).

To measure the effects of CXCR4 and CXCR7 overexpression on invasion in response to CXCL12 *in vivo*, we injected MTLn3 JP, MTLn3 CXCR4, MTLn3 CXCR7, and MTLn3 CXCR4-CXCR7 cell lines labeled with GFP into the fourth mammary fat pads of SCID mice and allowed the tumors to grow until they reached an average volume of 1,300 mm^3 ^for *in vivo *invasion analysis. FACS analysis of the primary tumors confirmed that overexpression of CXCR4 and CXCR7 in the respective cell lines was conserved *in vivo *[see Additional data file 3]. Needles containing Matrigel and CXCL12 as a chemoattractant were inserted in the primary tumors and invasive cells collected for a four-hour period. Overexpression of CXCR4 dramatically increased the *in vivo *invasive behavior of MTLn3 cells to CXCL12, with peak invasion shifted to lower concentrations (Figure 2b). CXCR7 overexpression alone, however, did not enhance the ability of MTLn3 cells to invade in response to CXCL12, as they failed to invade *in vivo *at the highest concentration of CXCL12 (62.5 nM) tested compared with buffer levels, a concentration which induced a three-fold increase in invasion above background in the MTLn3 JP tumors. Similarly, expression of CXCR7 in the presence of CXCR4 also impaired the invasive response to CXCL12 at all concentrations, with the peak response at 15.6 nM being significantly reduced in the MTLn3 CXCR4-CXCR7 tumors (*P *< 0.005). Invasion in response to 6.25 nM CXCL12 was not tested for the MTLn3 CXCR7 strain since responses to the higher concentrations were similar to the buffer response and the *in vitro *chemotaxis data did not demonstrate any response at lower concentrations. These results are consistent with the *in vitro *invasive behavior of these cell lines, confirming that CXCR7 plays a negative role in CXCL12-induced invasion. The relatively weak *in vivo *invasive response seen at 15.6 nM CXCL12 in the MTLn3 CXCR4-CXCR7 cell line is significantly impaired upon addition of the CXCR4 inhibitor AMD3100 (*P *< 0.05; Figure 2c), indicating the remaining response is still mediated by CXCR4. As there is no increase in CXCR4 expression seen in the MTLn3 JP tumor cells [see Additional data file 3], the response seen in MTLn3 JP tumors to high levels of CXCL12 may reflect initiation of the paracrine loop by tumor-associated macrophages, which express CXCR4 [44-46].

These data show that CXCR7 expression alone plays no role in CXCL12-induced motility (chemotaxis or invasion). However, CXCR7 expression in the context of high levels of CXCR4, while enhancing chemotaxis to CXCL12, impairs invasion *in vitro *and *in vivo *to CXCL12. These results raised the possibility that CXCR7 inhibits the ability to degrade extracellular matrix in response to CXCL12 stimulation and hence invasion. To address this, we measured the ability of MTLn3 CXCR4 and MTLn3 CXCR4-CXCR7 cells to degrade fluorescently labeled matrix (Figure 2d). Although there was no significant difference in the ability of these cell lines to degrade matrix in the absence of stimulation, when exposed to CXCL12 only the CXCR4 overexpressors showed significantly increased degradation (*P *< 0.05), while the double overexpressors showed no increase, indicating that CXCR7 expression impairs CXCL12-induced invasion by suppressing CXCL12-induced matrix degradation. Evaluation of the expression of different MMPs (MMP1, MMP2, MMP3, MMP7, MMP9-14) at the mRNA level revealed that MMP12 was significantly higher in the CXCR4 line compared with the other lines after stimulation with CXCL12 (*P *< 0.01 by ANOVA) [see Additional data file 4]. Western blotting after CXCL12 stimulation also indicated that MMP12 expression was highest in the CXCR4 line [see Additional data file 4]. These results suggest that there is differential regulation of MMPs in the MTLn3 CXCR4 and CXCR4-CXCR7 cells upon CXCL12 stimulation with the CXCR4-expressing cells showing increased expression of MMPs such as MMP3, MMP10, and MMP12. Evaluation of breast cancer data present in the Oncomine database indicates that only MMP12 expression is significantly correlated with CXCR4 expression: from the nine breast cancer databases evaluated, the average correlation was 0.31 with an average *P *value for the correlation of less than 0.04. In the same databases, the average correlation of MMP12 with CXCR7 was -0.03, which was not significant (*P *< 0.46).

We had previously reported that CXCL12 could induce *in vivo *invasion in the MMTV-PyMT transgenic breast cancer model, and that this invasion was dependent on the EGF/CSF-1 paracrine loop between cancer cells and macrophages where CSF-1 is secreted by cancer cells and stimulates macrophages to produce EGF [21]. To test whether CXCL12 induced *in vivo *invasion in the MTLn3 CXCR4 model was also dependent on EGF/CSF-1 signaling, we tested the ability of these cells to invade *in vivo *in response to CXCL12 in the presence of either a neutralizing EGF antibody or a blocking CSF-1R antibody [40]. The MTLn3 CXCR4 invasive response to CXCL12 was significantly impaired in the presence of either antibody [see Additional data file 5], indicating EGF/CSF-1 signaling is required for CXCL12 induced *in vivo *invasion in this model. Thus CXCR4 overexpression did not override the dependency of these breast cancer cells on the EGF/CSF-1 paracrine loop for *in vivo *invasion.

### CXCR4 overexpression stimulates cancer cell motility within the primary tumor

**Figure 3:**
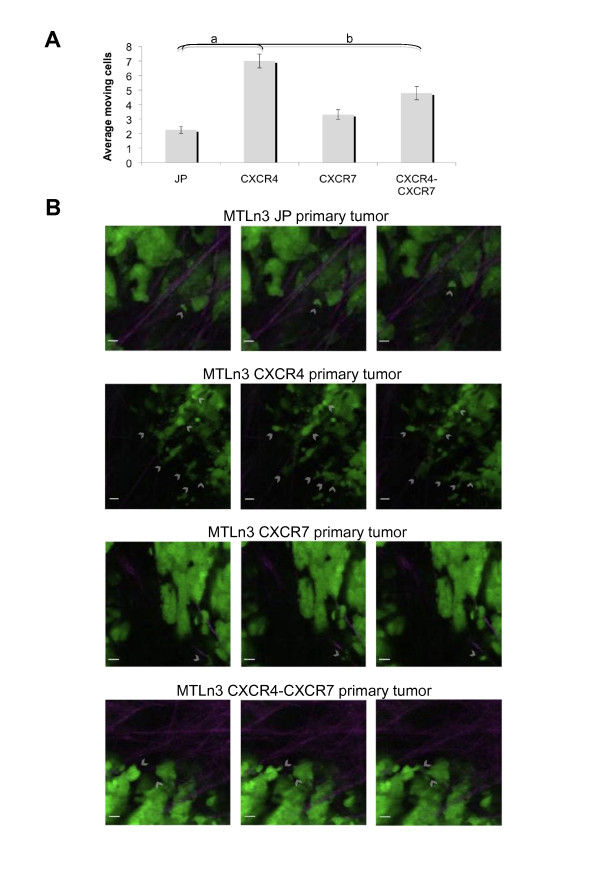
**Overexpression of CXCR4 enhances MTLn3 motility within the primary tumor**. GFP labeled MTLn3 transductants were orthotopically injected in the fourth mammary fat pad of SCID mice. Primary tumors with an average volume of 1,300 mm^3 ^were imaged using multiphoton microscopy. **(a) **Quantitation of the motility events observed in each tumor type. The average number of motile cancer cells (GFP positive) in a 50 μm Z-stack time-lapse series per movie is reported. Six MTLn3 JP animals were imaged with a total of 20 movies analyzed, four MTLn3 CXCR4 animals were imaged with 17 movies analyzed, four MTLn3 CXCR7 and four MTLn3 CXCR4-CXCR7 animals were imaged with 13 and 14 movies analyzed respectively. Means and SEMs are shown. MTLn3 JP vs. MTLn3 CXCR4 **(a) ***P *< 0.005; MTLn3 JP vs. MTLn3 CXCR4-CXCR7 **(b) ***P *< 0.05 with *P *values determined using Mann-Whitney. Scale bar: 10 μm. **(b) **Representative images of the motility observed in the indicated tumors, GFP tumor cells appear as green cells, host cells as dark shadows and ECM in purple. Individual frames are 10 minutes apart. Motile cancer cells are indicated by arrowheads.

Given the difference in the *in vitro *chemotactic and invasive behavior in response to CXCL12 stimulation of the double overexpressors, MTLn3 CXCR4-CXCR7, we proceeded to evaluate their motile behavior within the tumor microenvironment. The GFP labeled MTLn3 transductants were orthotopically injected in SCID mice and the tumors used for intravital imaging when they had reached an average volume of 1,300 mm^3^. Time-lapse z-series were taken using multiphoton microscopy to evaluate the number of cancer cells moving within the tumor microenvironment. Similar to the *in vitro *invasion results, CXCR4 overexpression significantly enhanced the motile behavior of MTLn3 cells within the primary tumor - about three-fold compared with MTLn3 JP cells (*P *< 0.005; Figure 3a). CXCR7 overexpression alone did not have a significant effect on cancer cell motility within the primary tumor compared with MTLn3 JP (*P *= 0.18). The double overexpressors, MTLn3 CXCR4-CXCR7, showed an intermediate phenotype that was greater than MTLn3 JP intravital motility (*P *< 0.05), but reduced compared with MTLn3-CXCR4 (*P *= 0.06). Representative images of cancer cell motility in each tumor are shown (Figure 3b) with representative movies included as Additional data files 6,7,8 to 9. In summary, the motile behavior of the different transductants *in vivo *most closely resembled their *in vitro *invasive response to CXCL12. Namely, while CXCR4 overexpression enhanced the motility of MTLn3 cells within the tumor, CXCR7 overexpression alone had no effect and in the context of CXCR4 overexpression, high levels of CXCR7 resulted in decreased motility. This suggests that despite the increased chemotactic response of the CXCR4-CXCR7 expressors to CXCL12 *in vitro*, their ability to invade extracellular matrix is impaired by their reduced degradation potential.

### CXCR7 overexpression enhances primary tumor growth while CXCR4 enhances intravasation

**Figure 4:**
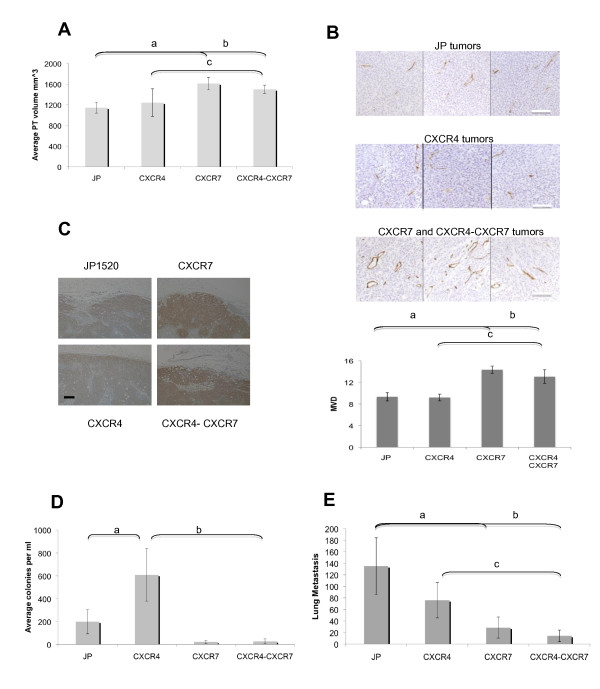
**Opposing roles of CXCR4 and CXCR7 in primary tumor growth, intravasation and lung metastasis formation**. **(a) **Primary tumor volumes at 30 to 34 days post orthotopic injection of MTLn3 JP (21 mice), MTLn3 CXCR4 (20 mice), MTLn3 CXCR7 (19 mice), and MTLn3 CXCR4-CXCR7 (29 mice). MTLn3 JP vs. MTLn3 CXCR7 **(a) ***P *< 0.05; MTLn3 JP vs. MTLn3 CXCR4-CXCR7 **(b) ***P *< 0.05; MTLn3 CXCR4 vs. MTLn3 CXCR4-CXCR7 **(c) ***P *< 0.05 with *P *values determined using Mann Whitney. **(b) **CXCR7 overexpression increases angiogenesis of MTLn3 tumors. MTLn3 JP, MTLn3 CXCR4, MTLn3 CXCR7, and MTLn3 CXCR4-CXCR7 primary tumors were harvested when they reached an average volume of 1,100 mm^3^, fixed and paraffin embedded. Immunohistochemistry to stain blood vessels was done using an antimouse CD34 antibody. Three tumors were used per cell line, with five fields counted per tumor. Representative images are shown (top), scale bar 100 μm, with the respective quantitation of mean vessel density (MVD, bottom). Student's t-test was used for two-condition comparisons and confirmed with ANOVA; MTLn3 JP vs. MTLn3 CXCR7 **(a) ***P *< 0.005; MTLn3 JP vs. MTLn3 CXCR4-CXCR7 **(b) ***P *< 0.05; MTLn3 CXCR4 vs. MTLn3 CXCR4-CXCR7 **(c) ***P *< 0.005. VEGFA immunohistochemistry of MTLn3 JP, MTLn3 CXCR4, MTLn3 CXCR7, and MTLn3 CXCR4-CXCR7 primary tumors. Three tumors were used per cell line with one representative tumor being shown, scale bar 200 μm. Staining in all samples is significantly greater than that seen with control staining with a nonspecific primary antibody (data not shown). Scale bar: 200 μm. **(d) **Intravasation of the different MTLn3 transductants measured using a blood burden assay. The total number of cancer cell colonies present were counted and normalized to the total volume of blood collected. MTLn3 JP *n *= 21 mice, MTLn3 CXCR4 *n *= 20 mice, MTLn3 CXCR7 *n *= 10 mice, MTLn3 CXCR4-CXCR7 *n *= 17 mice. MTLn3 JP vs. MTLn3 CXCR4 **(a) ***P *< 0.05; MTLn3 CXCR4 vs. MTLn3 CXCR4-CXCR7 **(b) ***P *< 0.005 with *P *values determined using Mann-Whitney. **(e) **Spontaneous lung metastasis formation of the MTLn3 transductants. The different cell lines were injected into the mammary fat pad and lungs harvested once the primary tumors reached an average volume of 1,500 mm^3^. MTLn3 JP *n *= 19 mice, MTLn3 CXCR4 *n *= 20 mice, MTLn3 CXCR7 *n *= 16 mice, MTLn3 CXCR4-CXCR7 *n *= 29 mice. The total number of lung metastases present in all lung lobes of a single H&E section are reported. MTLn3 JP vs. MTLn3 CXCR7 **(a) ***P *< 0.005; MTLn3 JP vs. MTLn3 CXCR4-CXCR7 **(b) ***P *< 0.005; MTLn3 CXCR4 vs. MTLn3 CXCR4-CXCR7 **(c) ***P *< 0.005. Means and SEMs are shown.

As CXCR4 and CXCR7 have been found to play a role in breast cancer growth and metastasis, we tested the effects of CXCR4 and CXCR7 overexpression on primary tumor growth, intravasation and lung metastasis formation of MTLn3 cells. Comparison of the volumes of MTLn3 CXCR4 tumors to the control MTLn3 JP tumors showed no difference (*P *= 0.38; Figure 4a), indicating that CXCR4 overexpression does not enhance growth of MTLn3 tumors. On the other hand, MTLn3 CXCR7 tumors showed significantly increased tumor size compared with the control MTLn3 JP tumors (*P *< 0.05), and similarly the MTLn3 CXCR4-CXCR7 double overexpressors were significantly bigger than either MTLn3 JP or MTLn3 CXCR4 tumors (*P *< 0.05); indicating that CXCR7 but not CXCR4 plays a role in enhancing tumor growth. Comparison of sizes of CXCR7 and CXCR4 tumors indicated a trend towards significance (*P *< 0.062). MTLn3 cells expressing CXCR7 alone or coexpressed with CXCR4-formed tumors that were visible macroscopically by day 12, while the control JP and CXCR4 expressing tumors were not visible until day 15 (data not shown). Importantly, we did not see a difference in the growth of these cell lines *in vitro *(data not shown). However, in line with previous reports [23,27], we observed increased blood vessel density in MTLn3 CXCR7 and MTLn3 CXCR4-CXCR7 primary tumor sections compared with control MTLn3 JP (*P *< 0.005) and MTLn3 CXCR4 tumors (*P *< 0.05), respectively (Figure 4b) using a CD34 antibody, suggesting that CXCR7 overexpression increases the growth of the primary tumor by stimulating angiogenesis. MTLn3 CXCR4 tumors showed blood vessel density comparable with that of MTLn3 JP tumors (*P *= 0.9). Previous studies have shown that CXCR7 expression can stimulate angiogenesis by inducing the secretion of VEGF [23,27]. Consistent with those studies, we found that VEGFA (Vascular endothelial growth factor A) expression is increased in MTLn3 CXCR7 and CXCR4-CXCR7 primary tumors compared to either MTLn3 JP or CXCR4 tumors (Figure 4c).

We next evaluated the effects of CXCR4 and CXCR7 overexpression on the process of intravasation, by doing intracardiac punctures prior to euthanization of mice carrying tumors of an average volume of 1,500 mm^3^. As shown in Figure 4d, MTLn3-CXCR4 cells were significantly more efficient in entering the bloodstream compared with MTLn3-JP and CXCR4-CXCR7. Both MTLn3 CXCR7 and MTLn3 CXCR4-CXCR7 tumor models showed low numbers of intravasated cancer cells. These results agree with the increased invasive and motile behavior of the CXCR4 overexpressing MTLn3 cells seen within the primary tumor, and the impaired ability of the CXCR7 overexpressors to invade *in vivo*.

To compare the ability of the MTLn3 transductants to spontaneously metastasize from the primary tumor to the lung, we evaluated lung metastasis formation in animals that had been injected in the mammary fat pad with the transduced MTLn3 cells. Lungs were harvested when the primary tumors reached an average volume of 1,500 mm^3^, fixed, paraffin-embedded and sectioned to count the number of metastases present per H&E section. The number of spontaneous lung metastases present in the MTLn3 CXCR4 model was not significantly different to the number of lung metastases formed in the empty vector control model, MTLn3 JP (*P *= 0.3 student's t-test and *P *= 0.8 Mann-Whitney; Figure 4e), nor was there any difference in the size of the metastases (data not shown). This was an unexpected result as MTLn3 CXCR4 cells were more efficient in leaving the primary tumor and entering the bloodstream compared with MTLn3 JP cells, thus we looked for metastasis formation in the bone and lymph nodes but no difference was observed at these sites (*P *= 0.7 and *P *= 0.8, respectively) [see Additional data file 10]. However, the MTLn3 CXCR7 tumors gave rise to significantly fewer lung metastases compared with the empty vector model, MTLn3 JP, (a, *P *< 0.005) and likewise, the double overexpressors, MTLn3 CXCR4-CXCR7, showed decreased lung metastasis compared with both MTLn3 JP (b, *P *< 0.005) and MTLn3 CXCR4 (c, *P *< 0.005; Figure 4e). No significant difference in size of lung metastasis, or in bone or lymph node metastasis was observed in the different CXCR7 transductants compared with control MTLn3 JP or MTLn3 CXCR4 (data not shown) [see Additional data file 10].

## Discussion

Previous studies have reported that both CXCR4 and CXCR7 play roles in breast cancer growth and metastasis, with both receptors being implicated in primary tumor growth, invasion and metastasis formation [2,6,10,12,22]. However, distinguishing the roles of these receptors in the early steps of metastasis has not been performed. Using the rat mammary adenocarcinoma cell line MTLn3, we studied the effects of overexpression of CXCR4 and CXCR7 on CXCL12-induced chemotaxis and invasion, as well as *in vivo *motility, intravasation and metastasis formation. We show that CXCR4 overexpression increases chemotactic and invasive behavior, both *in vitro *and *in vivo*, in response to a CXCL12 gradient, as well as enhances the motile behavior of tumor cells within the primary tumor and their ability to intravasate. Expression of CXCR7 alone had no effect on chemotaxis or invasion *in vitro*, but suppressed CXCL12-induced invasion *in vivo*, as well as intravasation and metastasis. Expression of both CXCR4- and CXCR7-enhanced chemotaxis to CXCL12 *in vitro*, but CXCL12-induced invasion *in vivo *and *in vitro *was reduced compared with that of cells expressing CXCR4 alone, and metastasis was also reduced.

The increased chemotactic response seen *in vitro *upon expression of CXCR4 is consistent with many previous studies demonstrating that CXCR4 can mediate chemotaxis to CXCL12 [47]. The literature on the ability of CXCR7 to mediate chemotactic responses is mixed, with some reports suggesting that CXCR7 can mediate chemotactic responses [1] and others indicating that it cannot [2,29]. Our data are consistent with the latter studies; using two cell lines (MTLn3 and MDA-MB-435) that show little chemotactic response to CXCL12 on their own, we find that expression of CXCR7 alone does not enhance chemotactic responses to CXCL12. However, coexpression of CXCR7 and CXCR4 resulted in increased chemotaxis towards CXCL12 compared with cells expressing CXCR4 alone. These data agree with a recent study showing that increased expression of CXCR7 in MDA-MB-231 cells results in enhanced chemotaxis to CXCL12 [36]. AMD3100, a CXCR4 selective inhibitor, inhibited CXCL12-induced chemotaxis and invasion of both MTLn3 CXCR4 and MTLn3 CXCR4-CXCR7 cells, while inhibition of CXCL12 binding to CXCR7 using I-TAC, CCX733 or CCX771 had no effect on CXCL12-induced chemotaxis. This indicates that CXCL12 binding to CXCR4 is needed for the chemotactic response but that binding of CXCL12 to CXCR7 is not necessary. The potentiation of the chemotactic response by CXCR7 is potentially through regulation of downstream signaling by CXCR4. CXCR7 has been shown to heterodimerize with CXCR4 [33-35] and to regulate recruitment of β-arrestin 2, as well as enhance ERK and p38 signaling in response to CXCL12 stimulation [36,48]. Our results suggest that although CXCR7 has been shown to alter G_αi _coupling to CXCR4 [34], the enhancement of β-arrestin signaling by CXCR7 [36] is more significant, resulting in enhanced chemotactic responses. It has been shown that CXCR7 can act as a scavenger receptor that internalizes CXCL12 and in that way decreases binding of CXCL12 to CXCR4 [49,50], therefore downregulating CXCR4 signaling. This has been proposed as a mechanism for suppression of chemotaxis to CXCL12 at low concentrations of CXCL12 [34]. Under our chemotaxis conditions, the double overexpressors showed increased chemotactic behavior *in vitro *to CXCL12 compared with the CXCR4 overexpressors even at low CXCL12 concentrations, suggesting that the scavenging function was not reducing chemotaxis *in vitro*. It is possible that under our *in vitro *conditions (in which there is a large volume in the attractant well, which is unlikely to be depleted during the time scale of the experiment) the scavenging function could actually increase chemotactic responses by reducing the amount of CXCL12 that leaks past the cells into the buffer side, and thereby maintaining a steeper gradient [51].

However, we found that coexpression of CXCR7 with CXCR4 did impair CXCL12-induced invasion *in vitro *of MTLn3 CXCR4 cells. CXCR7 potentially could modulate CXCR4 regulated gene expression by signaling through β-arrestin 2 [30,32,34]. This might result in decreased ability to degrade extracellular matrix, which could translate into a defect in invasion but not chemotaxis. Indeed this seems to be the case as the double overexpressors showed significantly reduced matrix degradation in response to CXCL12 treatment compared with the CXCR4 overexpressors. Evaluation of MMP expression in the MTLn3 transductants showed increased MMP12 mRNA expression upon CXCL12 stimulation in the MTLn3 CXCR4 cell line compared with the other transductants including the CXCR4-CXCR7 double expressor. Although MMP3 has been reported to be induced by CXCR7 [52], we did not observe that in the MTLn3 lines. Examination of breast cancer studies in the Oncomine database supports the possibility that CXCR4 can regulate MMP12 expression: MMP12 expression correlated significantly with CXCR4 expression, and not with CXCR7 expression. In summary, we propose that stimulation of CXCR4 can induce expression of MMP12 to increase invasiveness, and simultaneous expression of CXCR7 may suppress this induction.

The control cell line MTLn3 JP, although failing to chemotax or invade *in vitro *in response to CXCL12 stimulation, showed enhanced invasion *in vivo *at high concentrations of CXCL12. In addition, expression of CXCR7 alone reduced the invasion seen at high concentrations of CXCL12. As MTLn3 JP did not show chemotaxis to CXCL12 at any concentration *in vitro *and we did not observe upregulation of CXCR4 expression *in vivo *in this control cell line, we believe that the *in vivo *invasion at high concentration is a result of stimulating macrophages (which express CXCR4 [44-46]) within the tumor microenvironment, which can promote cancer cell invasion through the paracrine loop [21]. The reduced *in vivo *invasion of the CXCR7 expressing cells could be due to the scavenger function of CXCR7. For the *in vivo *invasion assay, CXCL12 diffusion would be constrained in the compact microenvironment to the spaces between cells. CXCR7 expressed on the tumor cells could then scavenge CXCL12, resulting in suppression of the activation of the invasion response.

The CXCR4 overexpressing line showed a strong *in vivo *invasion response to CXCL12, consistent with its strong chemotaxis and invasion responses *in vitro*. The invasion response was mediated by the paracrine loop with macrophages, as demonstrated by inhibition of invasion by either blocking EGF or CSF1R signaling. The CXCR4-CXCR7 line showed a reduced *in vivo *invasion response, which could reflect a CXCR7-induced reduction in matrix degradation (as we demonstrated for *in vitro *invasion), scavenging of CXCL12 by CXCR7, or both.

Consistent with previous reports, overexpression of CXCR7 resulted in a small but statistically significant increase in primary tumor growth possibly due to increased angiogenesis [22]. This was correlated with increased microvessel density and increased VEGFA expression. VEGFA has been shown to be upregulated by CXCR7 in a number of tumor cells [23,27,53], and thus it is likely that the increase in VEGF in the CXCR7 expressing lines leads to increased angiogenesis and tumor growth. We did not see an effect of CXCR4 expression on primary tumor growth as previously reported in other breast cancer models [11,12], suggesting that this effect might be cell line specific.

The intravasation efficiencies of the various lines correlated with their *in vivo *invasiveness, with the CXCR4 overexpressing lines showing significantly more intravasation than the other lines. This suggests that CXCL12 signaling could contribute to the intravasation process. Indeed, perivascular macrophages have been shown to express CXCL12 [54], and thus a local gradient of CXCL12 leading towards blood vessels could stimulate directed invasion around vessels. Thus the CXCR7 lines, which show reduced invasion to CXCL12 gradients, would also be reduced in their intravasation capability.

A paradoxical result is our finding that CXCR4 overexpression did not result in increased spontaneous lung metastasis formation despite enhancing invasion and intravasation. This result disagrees with previous studies suggesting that wild-type or mutant CXCR4 mediates spontaneous metastasis of breast cancer cells to the lungs [6,55,56]. However, in those studies, CXCR4 signaling was shown to have a significant effect on primary tumor growth (or primary tumor size was not provided), and metastasis was compared at equal times rather than equal primary tumor sizes, leaving open the possibility that the increased metastasis seen in those studies reflects the increased intravasation from a larger primary tumor. Alternatively, it is possible that the effects of CXCR4 expression on metastasis varies with the particular model used, similar to the varying effects on growth. Our results cannot be explained by impaired cell survival of intravasated cancer cells since the intravasation assay used in this study evaluated viable cells. One possibility is that there might be gene expression changes occurring in MTLn3 CXCR4 cells within the tumor microenvironment that provide an advantage in entering the circulation but impair the ability to extravasate or seed lung metastases. Indeed, MMP12, which we find upregulated in CXCR4 cells stimulated with CXCL12, has been shown to be antiangiogenic [57-59]. Thus it is possible that although MMP12 is helpful in enabling tumor cells to invade, its antiangiogenic effects suppress the ability of tumor cells to extravasate and successfully seed metastases in the lung.

In summary, our studies provide insight into the complexity of the contributions of CXCR4 and CXCR7 to tumor cell invasion and metastasis. CXCR4 can enhance local invasion and intravasation due to CXCL12-induced chemotaxis and matrix degradation. The ligand scavenging function of CXCR7 may have contrasting effects on chemotaxis and invasion depending upon the diffusion constraints imposed upon CXCL12. CXCR7 can affect tumor growth through increased angiogenesis. We have identified MMP12 as a potential mediator of CXCR4-enhanced invasion, and further work will be needed to test its contributions to the different steps of metastasis.

## Conclusions

We have found that CXCR4 and CXCR7 in breast cancer cells can make distinct contributions to tumor malignancy. CXCR4 expression increases tumor cell invasiveness and motility. CXCR7 expression inhibits invasion and metastasis, potentially through suppression of CXCR4 induced expression of MMP12. However, CXCR7 expression can stimulate VEGFA expression, microvessel density, and primary tumor growth.

## Abbreviations

ANOVA: analysis of variance; BSA: bovine serum albumin; CAF: cancer-associated fibroblast; CSF1: colony stimulating factor 1; DAB: diaminobenzidine; DAPI: 4',6-diamidino-2-phenylindole; EDTA: ethylenediaminetetraacetic acid; EGF: epidermal growth factor; EPC: endothelial progenitor cells; ER: estrogen receptor; FACS: fluorescence-activated cell sorting; GAPDH: glyceraldehyde 3-phosphate dehydrogenase; GPCR: G-protein coupled receptor; H&E: hematoxylin and eosin; IHC: immunohistochemistry; I-TAC/CXCL11: interferon-inducible T-cell alpha chemoattractant; MAPK: mitogen-activated protein kinase; MMP: matrix metalloproteinase; PBS: phosphate buffered saline; PI3K: phosphatidylinositol 3-kinase; RT-PCR: reverse transcription polymerase chain reaction; SCID: severe combined immunodeficiency; VEGF: vascular endothelial growth factor.

## Competing interests

The authors declare that they have no competing interests.

## Authors' contributions

LH generated the cell lines, performed the *in vitro *chemotaxis, invasion and FACS analyses as well as all the *in vivo *assays and contributed to the writing of the manuscript. MM and JC performed the *in vitro *analysis of matrix degradation. SJC performed Western blotting for MMP12. JS contributed to the design of the experiments and writing of the manuscript. All authors read and approved the final manuscript.

## Supplementary Material

Additional file 1 **Table 1**. Mean fluorescence intensity values were obtained using FlowJo software with values for CXCR4 and CXCR7 expression normalized to the IgG isotype control for each cell line. Representative values are shown. Click here for file

Additional file 2 **(a) Endogenous levels of CXCR4 expression. FACS analysis of the MTLn3 transductants for rat CXCR4 expression using a rat specific antibody (dotted lines) and isotype control IgG (grey shaded peaks)**. (**b) **FACS analysis of the MDA MB 435 transductants for CXCR4 and CXCR7 expression with representative plots being shown. Isotype control mouse IgG (grey shaded peaks), anti-CXCR4 antibody (dashed lines, MAB172) and anti-CXCR7 antibody (dotted lines, 11G8). **(c) **Chemotaxis of the MDA MB 435 transductants to CXCL12 using a microchemotaxis chamber. Total number of cells per well are reported (11 to 39 wells were counted per condition). Comparison of 435 CXCR4 chemotaxis to CXCL12 with that of 435 JP cells shows a statistical significant increase at 1 nM and 5 nM with a *P *value < 0.05 and < 0.005 respectively, as determined by t-test. Comparison of the chemotaxis of the double expressors, CXCR4-CXCR7, with that of 435 JP cells shows similarly statistically significant differences as determined by t-test, with *P *< 0.005 at 1 nM and 5 nM CXCL12 and *P *< 0.05 at 25 nM. Statistical significant differences between 435 CXCR4 and 435 CXCR4-CXCR7 are indicated in the figure with *P *< 0.05 indicated by * and *P *< 0.005 indicated by **. Means and SEMs are shown. Click here for file

Additional file 3 **FACS analysis of CXCR4 and CXCR7 expression in MTLn3 cells isolated from primary tumors**. MTLn3 JP, MTLn3 CXCR4, MTLn3 CXCR7, and MTLn3 CXCR4-CXCR7 cell lines labeled with GFP were injected in the fourth mammary fat pad of female SCID mice. When tumors reached an average volume of 1,300 mm^3^, the tumors were harvested, mechanically disrupted and labeled with either a mouse anti-CXCR4 antibody (MAB172) or a mouse anti-CXCR7 antibody (11G8). Grey shaded peaks represent unlabeled primary tumor sample with the peaks in solid lines representing CXCR4 expression of the carcinoma cells and the peaks in dashed lines representing CXCR7 expression. Click here for file

Additional file 4 **Expression levels of MMPs in MTLn3 cell lines stimulated with CXCL12. (a) **The indicated MTLn3 cell lines were stimulated with CXCL12 as described in Methods and then the level of expression of the indicated MMP was determined by quantitative RT-PCR. For each experiment, the delta CT vs GAPDH was determined and then normalized to the MTLn3-CXCR4 value. Higher values correspond to lower levels of mRNA. The results for MMP2, MMP7, MMP9, and TIMP1 are single experiments which were not repeated because there was no indication of a difference between the cell lines. For MMP1, MMP3, MMP10, MMP12, MMP13, MMP14, and TIMP2, the data are means and SEMS of at least three measurements. **(b) **Western blotting of extracts of cells prepared as in a. using an anti-MMP12 antibody (Epitomics 1906-1). Click here for file

Additional file 5 **CXCL12 induced *in vivo *invasion in MTLn3 CXCR4 tumors requires the EGF/CSF-1 paracrine loop**. *In vivo *invasion of MTLn3 CXCR4 tumors to CXCL12 in the presence of either a control IgG antibody (IgG), a blocking CSF-1R antibody (CSF1R Ab) or a neutralizing EGF antibody (EGF Ab). At least three animals were tested with seven to eight needles counted per condition. Means and SEMs are shown. Student's t-test was used for comparisons, *P *< 0.05 is represented by * and *P *< 0.005 by **. Click here for file

Additional file 6 **Time lapse imaging of MTLn3 JP1520 empty vector control cells (GFP, green) with extracellular matrix fibers imaged using second harmonic scattering (purple)**. Frames were taken every two minutes, scale bar is 10 μm. Click here for file

Additional file 7 **Time lapse imaging of MTLn3 CXCR4 cells (GFP, green) with extracellular matrix fibers imaged using second harmonic scattering (purple)**. Frames were taken every two minutes, scale bar is 10 μm. Click here for file

Additional file 8 **Time lapse imaging of MTLn3 CXCR7 cells (GFP, green) with extracellular matrix fibers imaged using second harmonic scattering (purple)**. Frames were taken every two minutes, scale bar is 10 μm. Click here for file

Additional file 9 **Time lapse imaging of MTLn3 CXCR4-CXCR7 cells (GFP, green) with extracellular matrix fibers imaged using second harmonic scattering (purple)**. Frames were taken every two minutes, scale bar is 10 μm. Click here for file

Additional file 10 **CXCR4 expression does not increase metastasis of MTLn3 cells to the bone marrow or lymph nodes**. **(a) **Spontaneous bone metastasis formation. Bone marrow from the femur ipsilateral to the primary tumor was extruded into MTLn3 growth media and cancer colonies present a week after plating counted. The number of cancer colonies present per femur are reported (*P *= 0.69, Mann-Whitney). MTLn3 JP *n *= 21 mice, MTLn3 CXCR4 *n *= 30 mice. Means and SEMs are shown. **(b) **Axillary and inguinal lymph nodes were dissected from MTLn3 JP and MTLn3 CXCR4 tumor-bearing mice. Lymph nodes were fixed, paraffin embedded, sectioned and stained with H&E. The presence of metastases was assessed using a light microscope with 10× and 20× objectives. MTLn3 JP *n *= 13 mice, MTLn3 CXCR4 *n *= 10 mice, means and SEMs are shown, *P *= 0.8, Mann-Whitney. Click here for file
