# Supplementary material for: Opposing roles of CXCR4 and CXCR7 in breast cancer metastasis
Source: Breast Cancer Res. 2011 Dec 9;13(6):R128. doi: 10.1186/bcr3074 (PMC3326570; doi:10.1186/bcr3074)
Supplement: Additional file 1 — Table 1. Mean fluorescence intensity values were obtained using FlowJo software with values for CXCR4 and CXCR7 expression normalized to the IgG isotype control for each cell line. Representative values are shown. [file bcr3074-S1.DOCX]

**Table 1. Mean Fluorescence Intensity of CXCR4 and CXCR7 expression in MTLn3 and MDA-MB-435 transductants.** Mean fluorescence intensity values were obtained using FlowJo software with values for CXCR4 and CXCR7 expression normalized to the IgG isotype control for each cell line. Representative values are shown.

|  | **CXCR4** | **CXCR7** |
| --- | --- | --- |
| **MTLn3 JP1520** | 1326 | 115 |
| **MTLn3 CXCR4** | 10212 | 142 |
| **MTLn3 CXCR7** | 1664 | 657 |
| **MTLn3 CXCR4-CXCR7** | 10861 | 676 |
| **MDA MB 435 JP** | 407 | 41 |
| **MDA MB 435 CXCR4** | 9847 | 60 |
| **MDA MB 435 CXCR7** | 496 | 1359 |
| **MDA MB 435 CXCR4-CXCR7** | 9991 | 2219 |
