# Supplementary material for: Opposing roles of CXCR4 and CXCR7 in breast cancer metastasis
Source: Breast Cancer Res. 2011 Dec 9;13(6):R128. doi: 10.1186/bcr3074 (PMC3326570; doi:10.1186/bcr3074)

**Supplementary Figure 1**

**A**


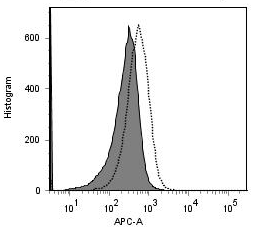

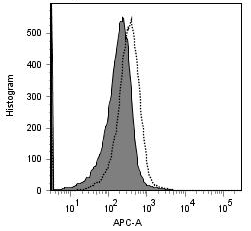

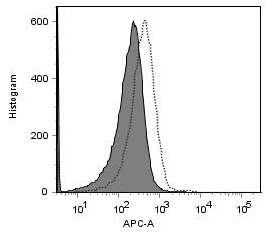

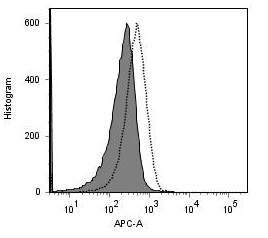


MTLn3 JP MTLn3 CXCR4 MTLn3 CXCR7 MTLn3 CXCR4-CXCR7

MDA MB 435 JP MDA MB 435 CXCR4 MDA MB 435 CXCR7 MDA MB 435 CXCR4- CXCR7

**B**


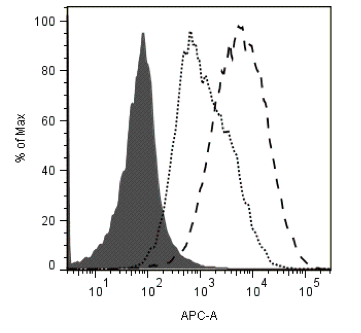

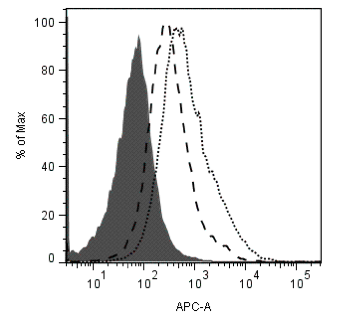

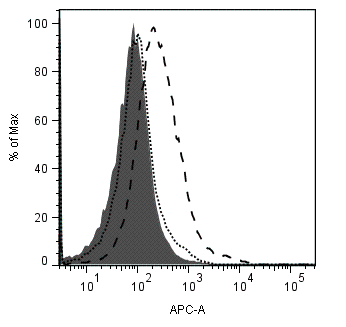

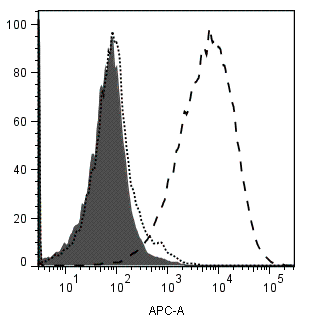


**
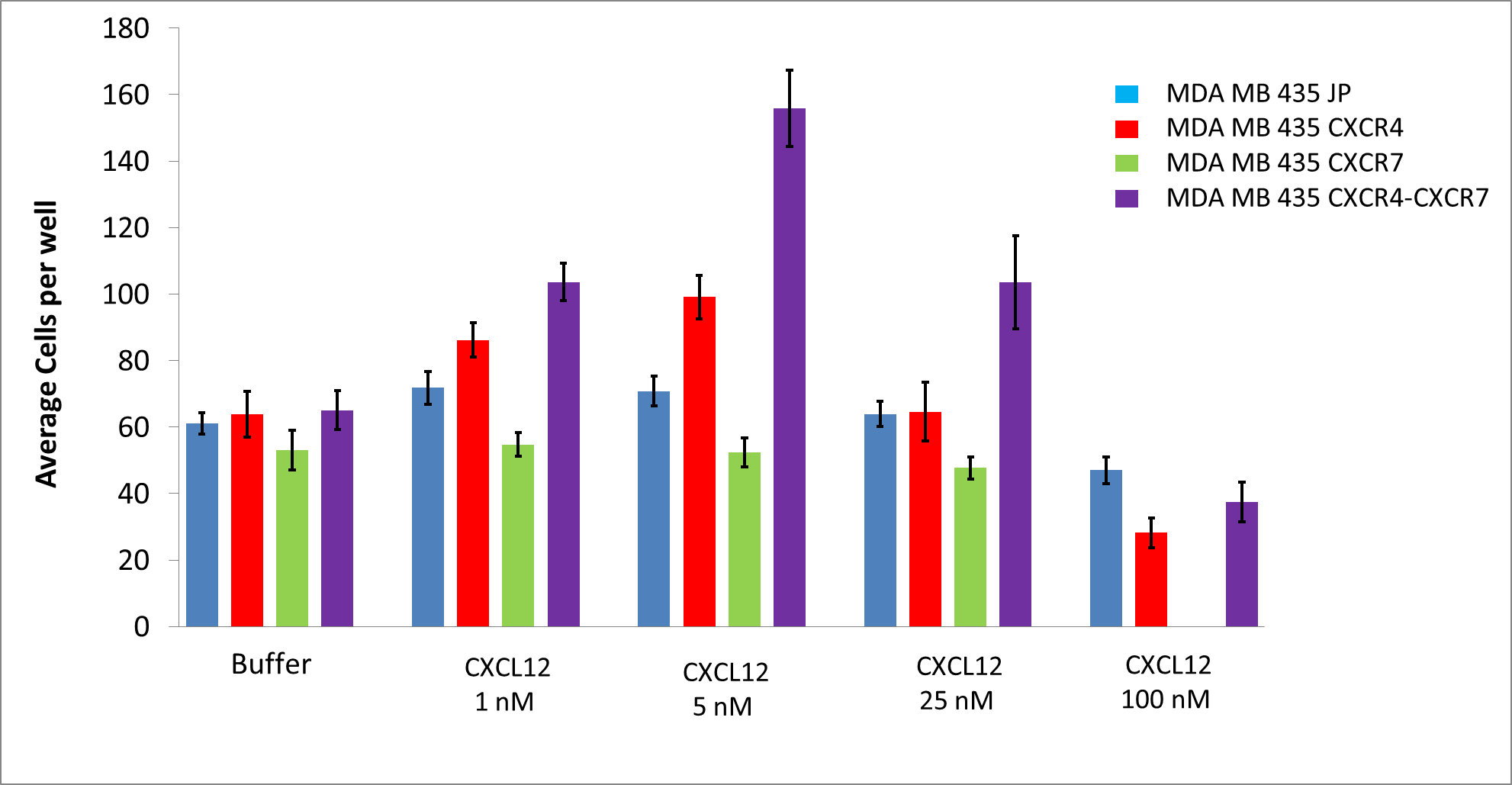
**

**


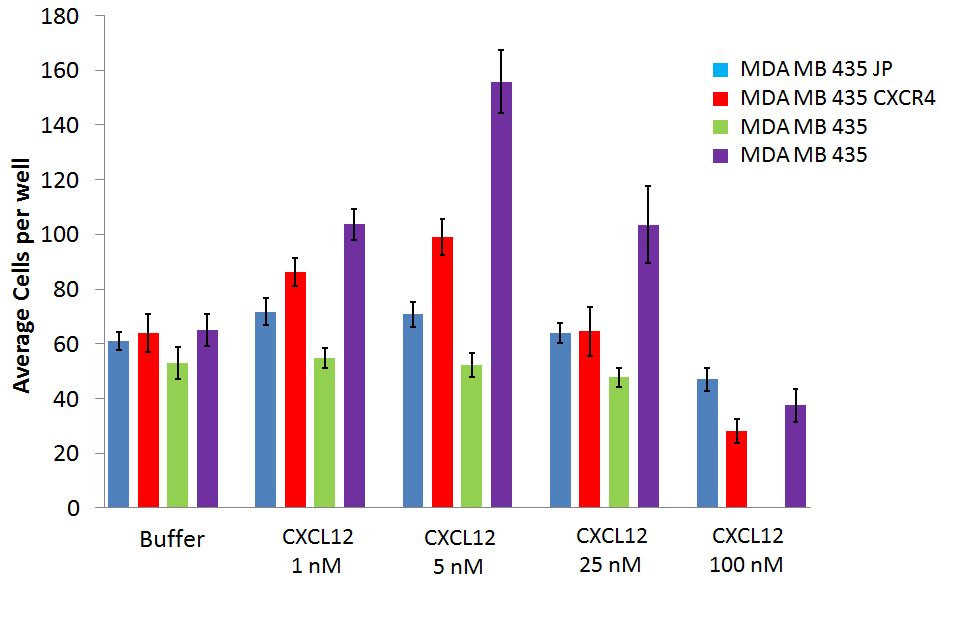


**C**

*

*


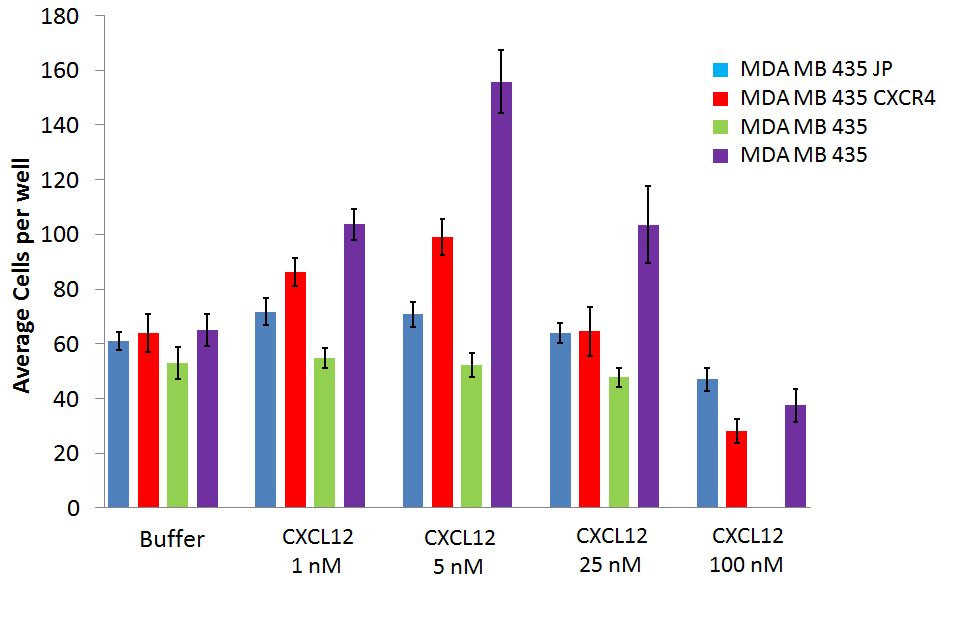

Supplement: Additional file 2 — (a) Endogenous levels of CXCR4 expression. FACS analysis of the MTLn3 transductants for rat CXCR4 expression using a rat specific antibody (dotted lines) and isotype control IgG (grey shaded peaks). (b) FACS analysis of the MDA MB 435 transductants for CXCR4 and CXCR7 expression with representative plots being shown. Isotype control mouse IgG (grey shaded peaks), anti-CXCR4 antibody (dashed lines, MAB172) and anti-CXCR7 antibody (dotted lines, 11G8). (c) Chemotaxis of the MDA MB 435 transductants to CXCL12 using a microchemotaxis chamber. Total number of cells per well are reported (11 to 39 wells were counted per condition). Comparison of 435 CXCR4 chemotaxis to CXCL12 with that of 435 JP cells shows a statistical significant increase at 1 nM and 5 nM with a P value < 0.05 and < 0.005 respectively, as determined by t-test. Comparison of the chemotaxis of the double expressors, CXCR4-CXCR7, with that of 435 JP cells shows similarly statistically significant differences as determined by t-test, with P < 0.005 at 1 nM and 5 nM CXCL12 and P < 0.05 at 25 nM. Statistical significant differences between 435 CXCR4 and 435 CXCR4-CXCR7 are indicated in the figure with P < 0.05 indicated by * and P < 0.005 indicated by **. Means and SEMs are shown. [file bcr3074-S2.DOC]
