# Supplementary material for: Opposing roles of CXCR4 and CXCR7 in breast cancer metastasis
Source: Breast Cancer Res. 2011 Dec 9;13(6):R128. doi: 10.1186/bcr3074 (PMC3326570; doi:10.1186/bcr3074)

**Supplementary Figure 2**

MTLn3 JP primary tumor MTLn3 CXCR4 primary tumor


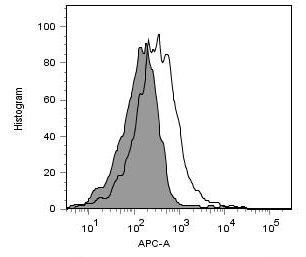


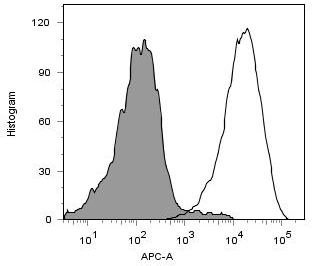


MTLn3 CXCR7 primary tumor MTLn3 CXCR4-CXCR7 primary tumor


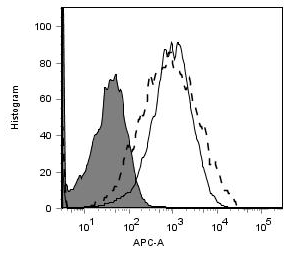

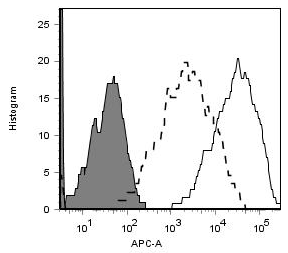

Supplement: Additional file 3 — FACS analysis of CXCR4 and CXCR7 expression in MTLn3 cells isolated from primary tumors. MTLn3 JP, MTLn3 CXCR4, MTLn3 CXCR7, and MTLn3 CXCR4-CXCR7 cell lines labeled with GFP were injected in the fourth mammary fat pad of female SCID mice. When tumors reached an average volume of 1,300 mm3, the tumors were harvested, mechanically disrupted and labeled with either a mouse anti-CXCR4 antibody (MAB172) or a mouse anti-CXCR7 antibody (11G8). Grey shaded peaks represent unlabeled primary tumor sample with the peaks in solid lines representing CXCR4 expression of the carcinoma cells and the peaks in dashed lines representing CXCR7 expression. [file bcr3074-S3.DOC]
