# Supplementary figures and images for: Opposing roles of CXCR4 and CXCR7 in breast cancer metastasis
Source: Breast Cancer Res. 2011 Dec 9;13(6):R128. doi: 10.1186/bcr3074 (PMC3326570; doi:10.1186/bcr3074)

**Supplementary Figure 3**

**A**

**B**


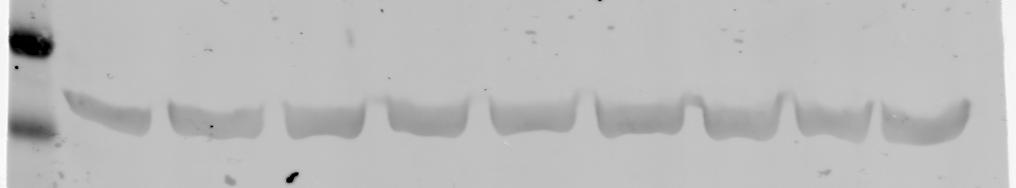

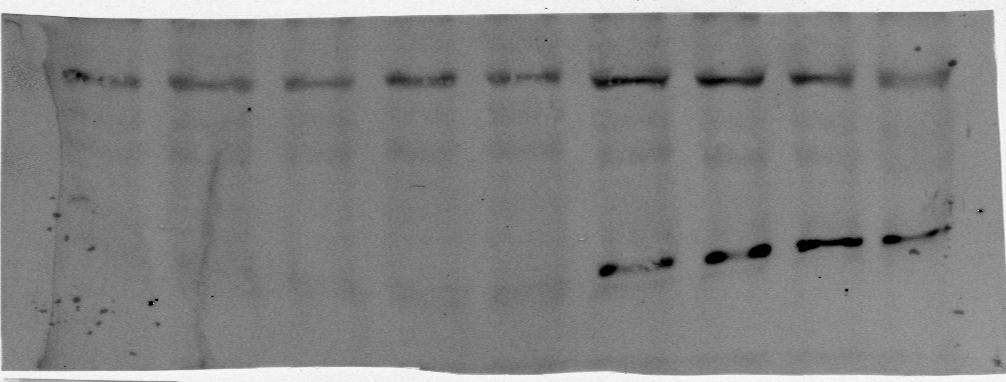


**JP1520 CXCR7 CXCR4 CXCR7/4**

Actin

MMP12

Supplement: Additional file 4 — Expression levels of MMPs in MTLn3 cell lines stimulated with CXCL12. (a) The indicated MTLn3 cell lines were stimulated with CXCL12 as described in Methods and then the level of expression of the indicated MMP was determined by quantitative RT-PCR. For each experiment, the delta CT vs GAPDH was determined and then normalized to the MTLn3-CXCR4 value. Higher values correspond to lower levels of mRNA. The results for MMP2, MMP7, MMP9, and TIMP1 are single experiments which were not repeated because there was no indication of a difference between the cell lines. For MMP1, MMP3, MMP10, MMP12, MMP13, MMP14, and TIMP2, the data are means and SEMS of at least three measurements. (b) Western blotting of extracts of cells prepared as in a. using an anti-MMP12 antibody (Epitomics 1906-1). [file bcr3074-S4.DOCX]

**Supplementary Figure 4**


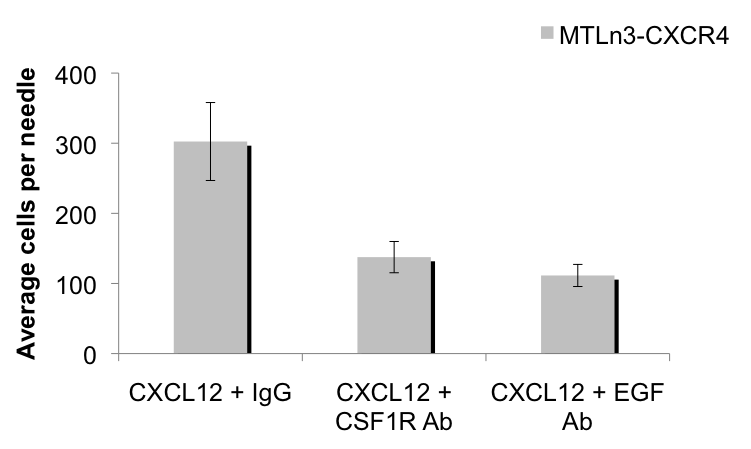


*

**

Supplement: Additional file 5 — CXCL12 induced in vivo invasion in MTLn3 CXCR4 tumors requires the EGF/CSF-1 paracrine loop. In vivo invasion of MTLn3 CXCR4 tumors to CXCL12 in the presence of either a control IgG antibody (IgG), a blocking CSF-1R antibody (CSF1R Ab) or a neutralizing EGF antibody (EGF Ab). At least three animals were tested with seven to eight needles counted per condition. Means and SEMs are shown. Student's t-test was used for comparisons, P < 0.05 is represented by * and P < 0.005 by **. [file bcr3074-S5.DOC]

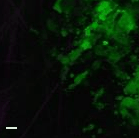

Supplement: Additional file 7 — Time lapse imaging of MTLn3 CXCR4 cells (GFP, green) with extracellular matrix fibers imaged using second harmonic scattering (purple). Frames were taken every two minutes, scale bar is 10 μm. [file bcr3074-S7.GIF]

**Supplementary Figure 5**

**A**

**B**

**
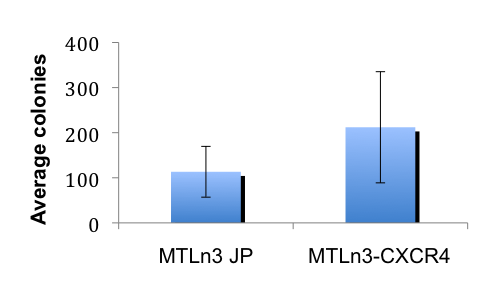

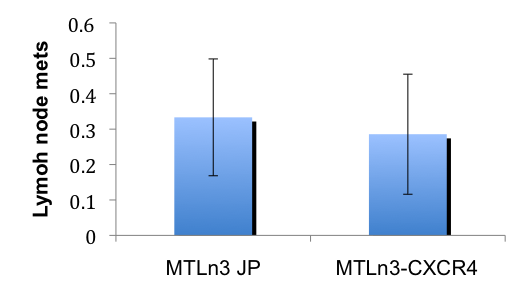
**

Supplement: Additional file 10 — CXCR4 expression does not increase metastasis of MTLn3 cells to the bone marrow or lymph nodes. (a) Spontaneous bone metastasis formation. Bone marrow from the femur ipsilateral to the primary tumor was extruded into MTLn3 growth media and cancer colonies present a week after plating counted. The number of cancer colonies present per femur are reported (P = 0.69, Mann-Whitney). MTLn3 JP n = 21 mice, MTLn3 CXCR4 n = 30 mice. Means and SEMs are shown. (b) Axillary and inguinal lymph nodes were dissected from MTLn3 JP and MTLn3 CXCR4 tumor-bearing mice. Lymph nodes were fixed, paraffin embedded, sectioned and stained with H&E. The presence of metastases was assessed using a light microscope with 10× and 20× objectives. MTLn3 JP n = 13 mice, MTLn3 CXCR4 n = 10 mice, means and SEMs are shown, P = 0.8, Mann-Whitney. [file bcr3074-S10.DOC]
